# Supplementary figures and images for: CardioTF, a database of deconstructing transcriptional circuits in the heart system
Source: PeerJ. 2016 Aug 23;4:e2339. doi: 10.7717/peerj.2339 (PMC5012272; doi:10.7717/peerj.2339)

A

TP:177

FP:9

FN:13

TN:11407

B

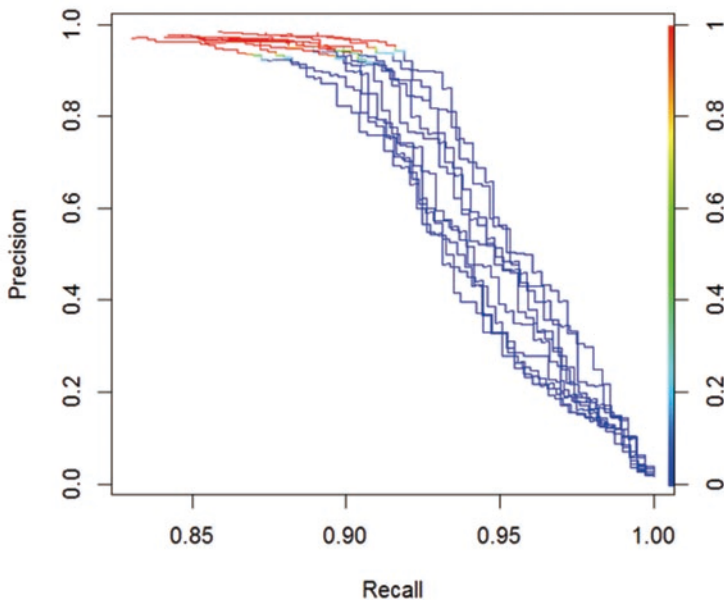

Supplement: Supplemental Information 4 — By convention, the class label of the minority class is positive (Weinstein abstracts), while the class label of the majority class is negative (non-Weinstein-like abstracts). (A) The confusion matrix for a two-class problem. The first column shows the actual class label of the examples, and the first row presents their predicted class label. In the matrix, TP shows the true positive samples, FP shows the false positive samples, TN shows the true negative samples, and FN shows the false negative samples. (B) The precision-recall curve. A 5 × 2 cross-validation approach was used to select the parameter. The ten curves in different colors indicate validation of the approach. [file peerj-04-2339-s004.pdf]

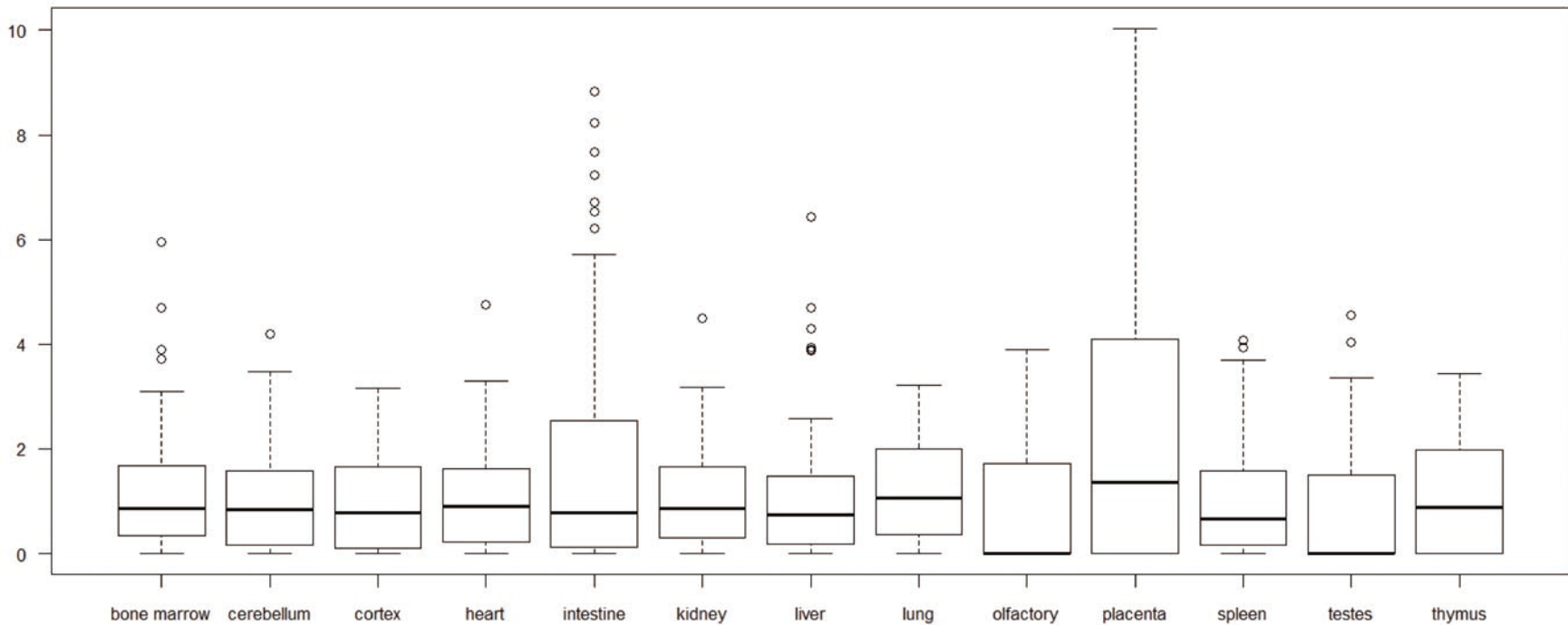

Supplement: Supplemental Information 7 — Expression level of RNA-seq data on the 81 TFs was plotted across different tissues. Nearly no difference can be ascertained across them at median level. [file peerj-04-2339-s007.pdf]

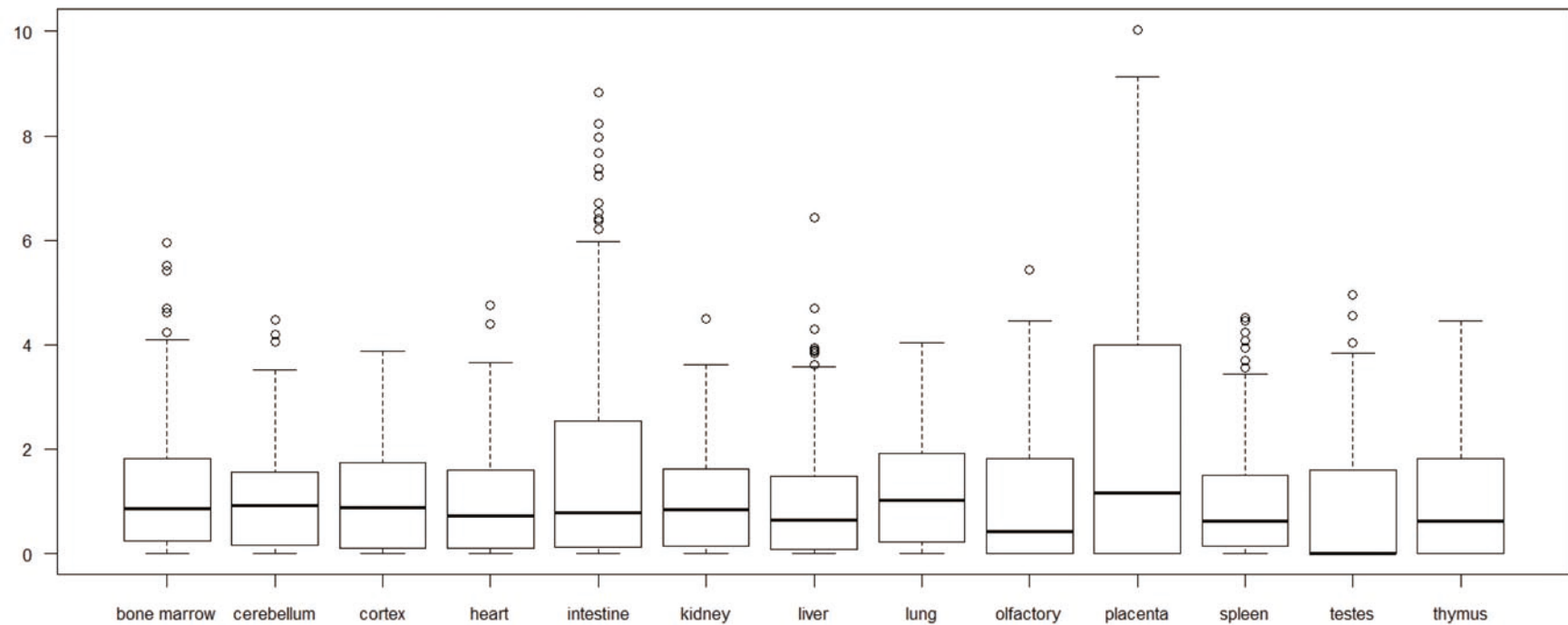

Supplement: Supplemental Information 8 — The expression level of those non-heart TFs across 13 adult tissues was plotted. Nearly all tissues have the same median expression level. [file peerj-04-2339-s008.pdf]

**Histone ChIP**  
**TF ChIP**

**Raw data**

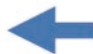

**Bowtie**

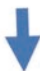

**MACS**

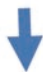

**Homer**

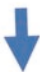

**MariaDB**

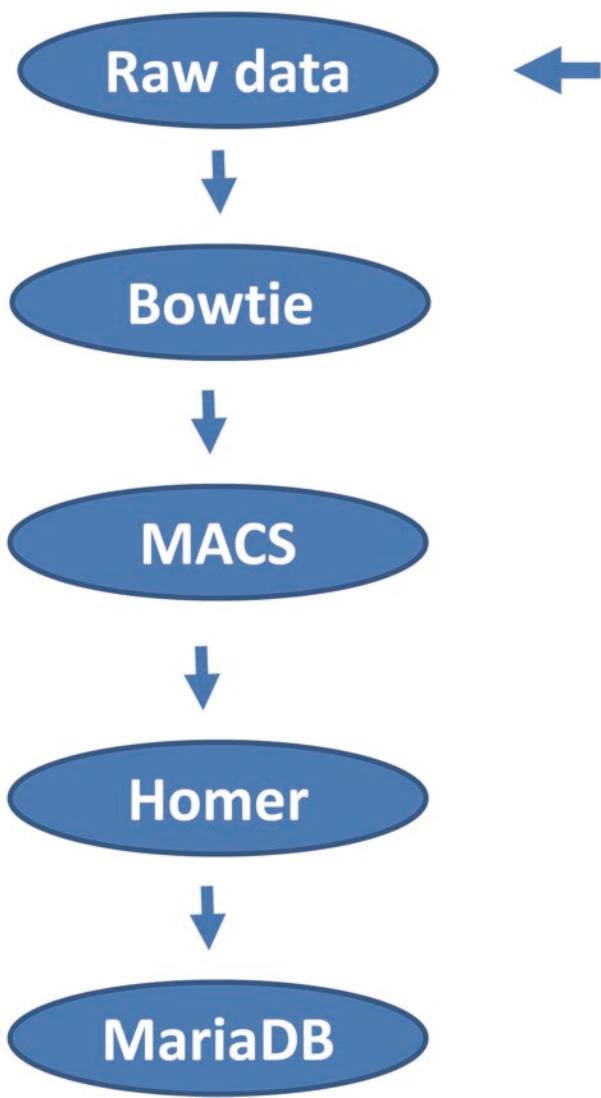

Supplement: Supplemental Information 9 — The raw data were downloaded from the public databases. After pre- and post-processing, the data were then analyzed with the analysis pipeline. The final results were dumped into the MySQL database at CardioTF. [file peerj-04-2339-s009.pdf]
