# Supplementary material for: CardioTF, a database of deconstructing transcriptional circuits in the heart system
Source: PeerJ. 2016 Aug 23;4:e2339. doi: 10.7717/peerj.2339 (PMC5012272; doi:10.7717/peerj.2339)
Supplement: Supplemental Information 5 — The results indicate that these TFs are truly associated with cardiac function by GO term enrichment analysis. Cluster 7 and Cluster 8 enriched TFs are involved in cardiac muscle differentiation and blood vessel development respectively, as evidenced by GO terms. Cardiac development includes the myocardium and vessel development which is in accordance with the analysis above. [file peerj-04-2339-s005.pdf]

Annotation Cluster 7	Enrichment Score: 12.99	G		Count	P_Value	Benjamini
GOTERM_BP_FAT	cardiac muscle tissue development	RT		14	7.2E-18	3.8E-16
GOTERM_BP_FAT	striated muscle tissue development	RT		16	6.0E-16	2.0E-14
GOTERM_BP_FAT	muscle tissue development	RT		16	1.8E-15	6.2E-14
GOTERM_BP_FAT	cardiac cell differentiation	RT		11	2.3E-15	7.9E-14
GOTERM_BP_FAT	cardiac muscle cell differentiation	RT		10	3.4E-14	1.0E-12
GOTERM_BP_FAT	muscle organ development	RT		16	8.5E-14	2.4E-12
GOTERM_BP_FAT	muscle cell differentiation	RT		13	3.4E-12	9.0E-11
GOTERM_BP_FAT	striated muscle cell differentiation	RT		10	2.3E-9	4.7E-8
GOTERM_BP_FAT	ventricular cardiac muscle cell differentiation	RT		6	2.8E-9	5.5E-8
**Annotation Cluster 8**	**Enrichment Score: 11.55**	**G**		**Count**	**P_Value**	**Benjamini**
GOTERM_BP_FAT	vasculature development	RT		19	4.1E-15	1.4E-13
GOTERM_BP_FAT	blood vessel morphogenesis	RT		17	2.7E-14	8.3E-13
GOTERM_BP_FAT	blood vessel development	RT		18	4.5E-14	1.3E-12
GOTERM_BP_FAT	angiogenesis	RT		8	1.3E-5	1.6E-4
