# Supplementary material for: CardioTF, a database of deconstructing transcriptional circuits in the heart system
Source: PeerJ. 2016 Aug 23;4:e2339. doi: 10.7717/peerj.2339 (PMC5012272; doi:10.7717/peerj.2339)
Supplement: Supplemental Information 6 — RNA-seq data of the 81 TFs at various developmental stages was clustered by hierarchical clustering using Pearson correlation distance. No pattern can be recognized across the 7 time points. [file peerj-04-2339-s006.pdf]

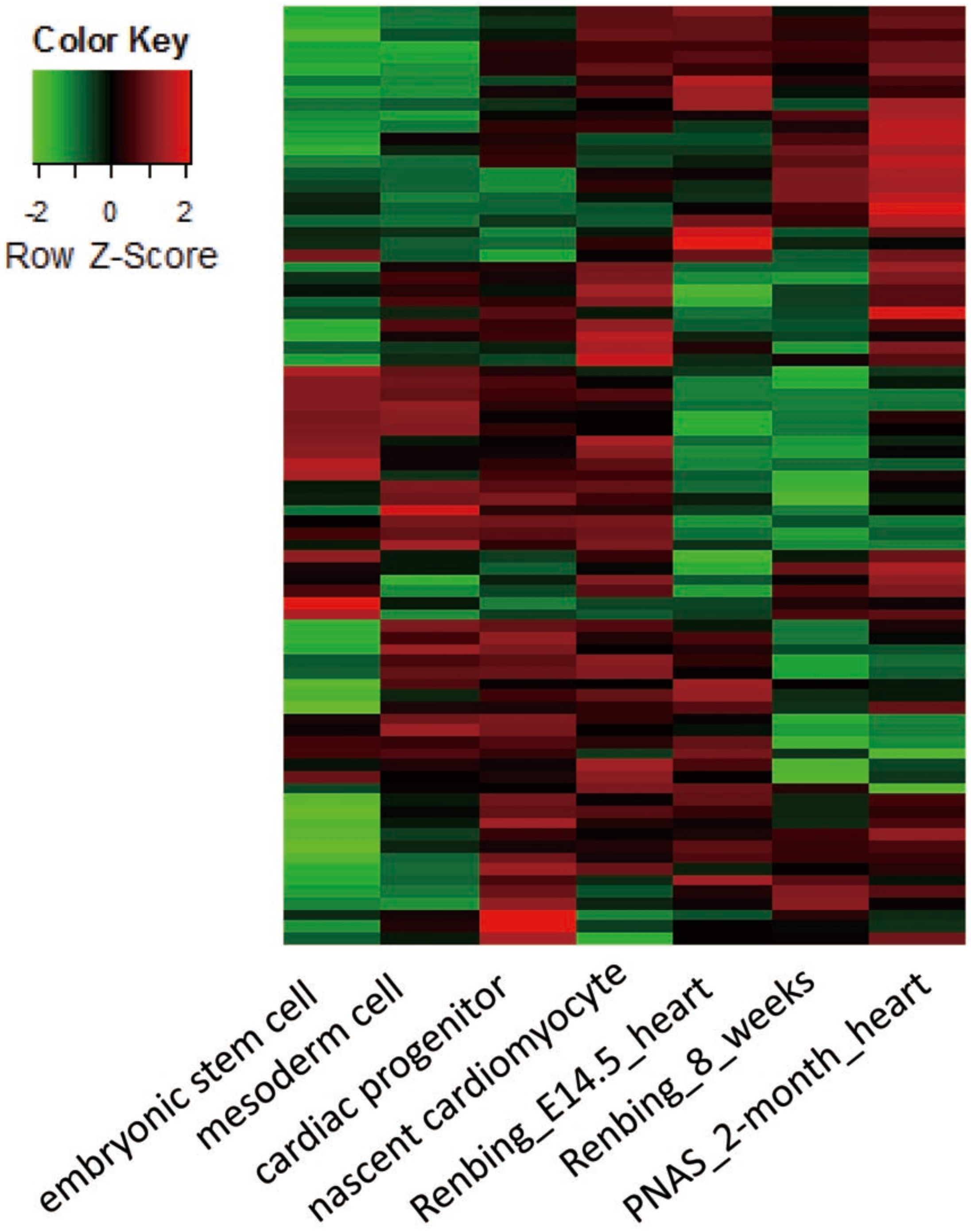

Color Key
-2
0
2
Row Z-Score
embryonic stem cell
mesoderm cell
cardiac progenitor
nascent cardiomyocyte
Renbing_E14.5_heart
Renbing_8_weeks
PNAS_2-month_heart
