# Supplementary material for: CardioTF, a database of deconstructing transcriptional circuits in the heart system
Source: PeerJ. 2016 Aug 23;4:e2339. doi: 10.7717/peerj.2339 (PMC5012272; doi:10.7717/peerj.2339)
Supplement: Supplemental Information 10 — These clusters focus on gene regulation as indicated by the presence of these GO terms. [file peerj-04-2339-s010.pdf]

			Count	P_Value	Benjamini
**Annotation Cluster 1**	**Enrichment Score: 64.02**		**Count**	**P_Value**	**Benjamini**
SP_PIR_KEYWORDS	transcription regulation	RT	74	9.3E-71	6.6E-69
SP_PIR_KEYWORDS	Transcription	RT	75	1.7E-68	6.1E-67
GOTERM_BP_FAT	transcription	RT	73	5.5E-55	1.2E-52
**Annotation Cluster 2**	**Enrichment Score: 60.6**		**Count**	**P_Value**	**Benjamini**
GOTERM_MF_FAT	DNA binding	RT	80	1.7E-68	4.1E-67
GOTERM_BP_FAT	regulation of transcription	RT	80	1.9E-61	6.9E-59
SP_PIR_KEYWORDS	nucleus	RT	80	5.1E-54	9.1E-53
**Annotation Cluster 3**	**Enrichment Score: 48.43**		**Count**	**P_Value**	**Benjamini**
GOTERM_BP_FAT	positive regulation of transcription	RT	50	1.9E-51	3.6E-49
GOTERM_BP_FAT	positive regulation of transcription, DNA-dependent	RT	48	6.4E-51	1.0E-48
GOTERM_BP_FAT	positive regulation of gene expression	RT	50	7.5E-51	1.0E-48
GOTERM_BP_FAT	positive regulation of RNA metabolic process	RT	48	9.1E-51	1.1E-48
GOTERM_BP_FAT	positive regulation of nucleobase, nucleoside, nucleotide and nucleic acid metabolic process	RT	50	6.9E-50	7.7E-48
GOTERM_BP_FAT	positive regulation of nitrogen compound metabolic process	RT	50	3.2E-49	3.3E-47
GOTERM_BP_FAT	positive regulation of macromolecule biosynthetic process	RT	50	4.7E-49	4.4E-47
GOTERM_BP_FAT	positive regulation of cellular biosynthetic process	RT	50	3.6E-48	3.1E-46
GOTERM_BP_FAT	positive regulation of biosynthetic process	RT	50	5.7E-48	4.5E-46
GOTERM_BP_FAT	positive regulation of transcription from RNA polymerase II promoter	RT	44	3.4E-47	2.6E-45
GOTERM_BP_FAT	positive regulation of macromolecule metabolic process	RT	50	3.3E-45	2.3E-43
