# Supplementary material for: CardioTF, a database of deconstructing transcriptional circuits in the heart system
Source: PeerJ. 2016 Aug 23;4:e2339. doi: 10.7717/peerj.2339 (PMC5012272; doi:10.7717/peerj.2339)
Supplement: Supplemental Information 11 — These clusters focus on gene regulation (cluster 4–5) and protein motifs (cluster 6). [file peerj-04-2339-s011.pdf]

Annotation Cluster 4	Enrichment Score: 24.76		Count	P_Value	Benjamini
GOTERM_CC_FAT	transcription factor complex	RT	26	2.1E-34	7.2E-33
GOTERM_CC_FAT	nucleoplasm part	RT	28	4.4E-29	7.8E-28
GOTERM_CC_FAT	nucleoplasm	RT	28	3.0E-27	3.5E-26
GOTERM_CC_FAT	nuclear lumen	RT	29	3.0E-24	2.6E-23
GOTERM_CC_FAT	intracellular organelle lumen	RT	29	2.8E-21	2.0E-20
GOTERM_CC_FAT	organelle lumen	RT	29	3.0E-21	1.8E-20
GOTERM_CC_FAT	membrane-enclosed lumen	RT	29	7.4E-21	3.7E-20
**Annotation Cluster 5**	**Enrichment Score: 15.86**		**Count**	**P_Value**	**Benjamini**
GOTERM_BP_FAT	negative regulation of transcription, DNA-dependent	RT	23	2.4E-18	1.4E-16
GOTERM_BP_FAT	negative regulation of RNA metabolic process	RT	23	2.8E-18	1.6E-16
GOTERM_BP_FAT	negative regulation of transcription	RT	24	9.0E-18	4.6E-16
GOTERM_BP_FAT	negative regulation of nucleobase, nucleoside, nucleotide and nucleic acid metabolic process	RT	24	3.8E-17	1.8E-15
GOTERM_BP_FAT	negative regulation of nitrogen compound metabolic process	RT	24	4.7E-17	2.2E-15
GOTERM_BP_FAT	negative regulation of transcription from RNA polymerase II promoter	RT	20	5.0E-17	2.2E-15
GOTERM_BP_FAT	negative regulation of macromolecule metabolic process	RT	26	5.2E-17	2.3E-15
GOTERM_BP_FAT	negative regulation of gene expression	RT	24	7.6E-17	4.6E-15
GOTERM_BP_FAT	negative regulation of macromolecule biosynthetic process	RT	24	1.2E-16	4.4E-15
GOTERM_BP_FAT	negative regulation of cellular biosynthetic process	RT	24	2.2E-16	8.5E-15
GOTERM_BP_FAT	negative regulation of biosynthetic process	RT	24	2.6E-16	8.3E-15
GOTERM_MF_FAT	transcription repressor activity	RT	14	3.5E-10	2.8E-9
**Annotation Cluster 6**	**Enrichment Score: 13.24**		**Count**	**P_Value**	**Benjamini**
SP_PIR_KEYWORDS	ubl conjugation	RT	22	6.8E-15	8.1E-14
SP_PIR_KEYWORDS	isopeptide bond	RT	17	6.6E-14	6.7E-13
UP_SEQ_FEATURE	cross-link:Glycyl lysine isopeptide (Lys-Gly) (interchain with G-Cter in SUMO)	RT	12	4.2E-13	3.6E-11
