# Supplementary material for: CardioTF, a database of deconstructing transcriptional circuits in the heart system
Source: PeerJ. 2016 Aug 23;4:e2339. doi: 10.7717/peerj.2339 (PMC5012272; doi:10.7717/peerj.2339)
Supplement: Supplemental Information 12 — These clusters indicate that TFs involved in other developmental process such as lung development and pattern specification are enriched. In Cluster 13, the TFs involved in epithelium development are enriched, which is in accordance with the epicardium development in heart. [file peerj-04-2339-s012.pdf]

Annotation Cluster	Term		Count	P_Value	Benjamini
**Annotation Cluster 9**	**Enrichment Score: 11.52**	G	**Count**	**P_Value**	**Benjamini**
GOTERM_BP_FAT	in utero embryonic development	RT	17	2.8E-12	7.5E-11
GOTERM_BP_FAT	chordate embryonic development	RT	20	2.9E-12	7.8E-11
GOTERM_BP_FAT	embryonic development ending in birth or egg hatching	RT	20	3.5E-12	8.8E-11
**Annotation Cluster 10**	**Enrichment Score: 10.78**	G	**Count**	**P_Value**	**Benjamini**
GOTERM_BP_FAT	tube development	RT	23	8.7E-20	5.4E-18
GOTERM_BP_FAT	respiratory system development	RT	11	2.7E-9	5.3E-8
GOTERM_BP_FAT	lung development	RT	10	1.7E-8	2.9E-7
GOTERM_BP_FAT	respiratory tube development	RT	10	2.0E-8	3.4E-7
**Annotation Cluster 11**	**Enrichment Score: 9.89**	G	**Count**	**P_Value**	**Benjamini**
GOTERM_BP_FAT	immune system development	RT	17	1.3E-11	3.0E-10
GOTERM_BP_FAT	hemopoietic or lymphoid organ development	RT	16	7.4E-11	1.7E-9
GOTERM_BP_FAT	hemopoiesis	RT	14	2.2E-9	4.6E-8
**Annotation Cluster 12**	**Enrichment Score: 8.87**	G	**Count**	**P_Value**	**Benjamini**
GOTERM_BP_FAT	pattern specification process	RT	17	7.1E-12	1.8E-10
GOTERM_BP_FAT	embryonic morphogenesis	RT	18	2.3E-11	5.3E-10
GOTERM_BP_FAT	tissue morphogenesis	RT	15	9.4E-11	2.1E-9
GOTERM_BP_FAT	regionalization	RT	13	4.0E-9	7.8E-8
GOTERM_BP_FAT	anterior/posterior pattern formation	RT	11	2.0E-8	3.5E-7
GOTERM_BP_FAT	gastrulation	RT	7	4.6E-6	6.5E-5
**Annotation Cluster 13**	**Enrichment Score: 7.82**	G	**Count**	**P_Value**	**Benjamini**
GOTERM_BP_FAT	tissue morphogenesis	RT	15	9.4E-11	2.1E-9
GOTERM_BP_FAT	epithelium development	RT	15	5.2E-10	1.1E-8
GOTERM_BP_FAT	morphogenesis of an epithelium	RT	8	7.0E-5	7.7E-4
